# Clinical management of nanophthalmos combined with cataract: a case series

**DOI:** 10.3389/fmed.2026.1827475

**Published:** 2026-05-14

**Authors:** Huiyu Zhu, Cun Sun, Renyue Xiao, Zhi Song, Xinjie Shu, Yu Gong, Jiawen Li

**Affiliations:** 1 Department of Ophthalmology, University-Town Hospital of Chongqing Medical University, Chongqing, China; 2 Department of Ophthalmology, West China Hospital, Sichuan University, Chengdu, Sichuan, China

**Keywords:** case series, cataract, clinical management, nanophthalmos, surgical treatment

## Abstract

**Objectives:**

To explore the clinical characteristics, surgical challenges, and management strategies for nanophthalmos combined with cataract.

**Case presentation:**

We report three adult patients with nanophthalmos, all presenting with extremely short axial lengths (Case 1: OD 14.83 mm, OS 14.54 mm; Case 2: OD 16.27 mm; Case 3: OD 15.19 mm, OS 15.00 mm) complicated by varying degrees of cataracts and other ocular complications. All three patients underwent phacoemulsification cataract extraction with intraocular lens implantation following admission.

**Conclusion:**

Patients with nanophthalmos face higher risks during cataract surgery compared to the general population, with a greater likelihood of postoperative complications such as uveal effusion syndrome, choroidal detachment, and a fibrotic membrane on the anterior IOL surface. Thorough perioperative assessment, meticulous surgical technique, individualized complication management, and long-term follow-up are critical to improving outcomes.

## 1 Introduction

Nanophthalmos is a developmental eye disorder characterized by a total axial length (AL) that is two standard deviations shorter than the average length for the same age group (1). This includes a general reduction in AL or a shortening of either the anterior or posterior segment of the eye (2, 3). The definition of AL for this condition remains unclear. Duke-Elder first introduced this concept, defining it as AL between 16.0 and 18.5 mm, and other studies define it as the distance from the corneal apex to the posterior scleral wall being less than 21 mm (4, 5). Additionally, some research defines the axial length of nano-eyes as less than 20.5 mm (6, 7), or less than 20 mm (8). This condition is associated with mutations in multiple genes including PRSS56, MFRP, and MYRF (9). Among Chinese affected families, nanophthalmos caused by mutations in the PRSS56, MFRP, and MYRF genes accounts for a significant proportion (9, 10). This category of eye diseases is influenced by both genetic and environmental factors, exhibiting high genetic heterogeneity (9). Their inheritance patterns include autosomal dominant inheritance, autosomal recessive inheritance, and X-linked inheritance, with recessive inheritance typically presenting more severe phenotypes (9, 11–14). This disease exhibits a complex clinical phenotype and is frequently associated with high hyperopia, angle-closure glaucoma (ACG), uveal effusion syndrome (UES), retinal detachment, and other conditions (9, 15, 16). This places such patients at a higher risk of intraoperative and postoperative complications during eye surgery (17, 18). Therefore, in surgical treatment for nanophthalmos combined with other ocular pathologies, effectively preventing and managing complications represents a key challenge in clinical practice. Among the three cases of nanophthalmos reported herein, one presented with an exceptionally short AL (14.54 mm)—a finding particularly rare among documented adult cases. This report aims to provide insights for the clinical management of such patients by analyzing the diagnostic and therapeutic course of these cases.

## 2 Case report

This case series includes five eyes from three patients diagnosed with nanophthalmos complicated by cataracts at Chongqing Medical University Affiliated University Town Hospital. Case 1 and Case 2 are members of the same family. Case 1 presented with bilateral involvement and developed complex complications including ACG, high hyperopia, UES, retinal detachment, and macular edema (ME). The patient underwent bilateral YAG laser iridotomy. Case 2 presents with right eye nanophthalmos, complicated by ACG, high hyperopia, and ME. The patient underwent right eye laser iridotomy. Both parents are deceased and reportedly had normal vision. Another sibling also has nanophthalmos but without glaucoma or other complications. No other family members exhibit disease. Case 3 presents with bilateral nanophthalmos, complicated by cataracts, optic disk crowding, and high hyperopia. The patient has a younger sister with poor vision since childhood (Table 1).

**TABLE 1:** Ocular characteristics of three patients.

Parameter	Case 1		Case 2		Case 3	
	OD	OS	OD	OS	OD	OS
UCVA	0.82	2.0	1.7	NLP	1.9	1.9
IOP	12 mmHg	14 mmHg	16 mmHg	T-2	17 mmHg	16 mmHg
AL	14.83 mm	14.54 mm	16.27 mm	—	15.19 mm	15.00 mm
ACD	Central 1.5 CT, peripheral < 1/4 CT.	Central 1.5 CT, peripheral < 1/4 CT.	Central 1.5 CT, peripheral < 1/4 CT.	Phthisis bulbi	Central 1 CT, peripheral < 1/4 CT.	Central 1 CT, peripheral < 1/4 CT.
CCD	519.5 μm	507.7 μm	534.5 μm	Conjunctival Overgrowth	—	—
ECC	3158.8/mm^2^	2879.1/mm^2^	1669.8 mm^2^	Conjunctival overgrowth	2909.1/mm^2^	2770.2/mm^2^
Conjunctiva	(−)	(−)	(+)	(+)	(−)	(−)
Sclera	(−)	(−)	(−)	—	(−)	(−)
Iris	Posterior synechiae, iridotomy at 9:00	Posterior synechiae, iris depigmentation changes at 6:00	Posterior synechiae, iridotomy at 11:00	—	Normal	Normal
Lens	Opacity (C2N3)	Opacity (C3N5)	Opacity	—	Opacity (C3N3)	Opacity (C3N3)
Pupil	No PLR	No PLR	IPS; temporal lateral light reflex present	—	Normal	Normal
Fundus	Foveal hypoplasia with crowded optic disks.	Foveal hypoplasia with crowded optic disks.	Macular oedema	—	Crowded optic disks	Crowded optic disks

All visual acuity measurements are provided in logMAR notation. UCVA, uncorrected visual acuity; IOP, intraocular pressure; AL, axial length; ACD, anterior chamber depth; CCD, central corneal thickness; ECC, endothelial cell count; IPS, irregular pupil shape; PLR, pupillary light reflex. (−) indicates no congestion, (+) indicates mild congestion, “—” indicates not applicable.

### 2.1 Case 1

**FIGURE 1:**
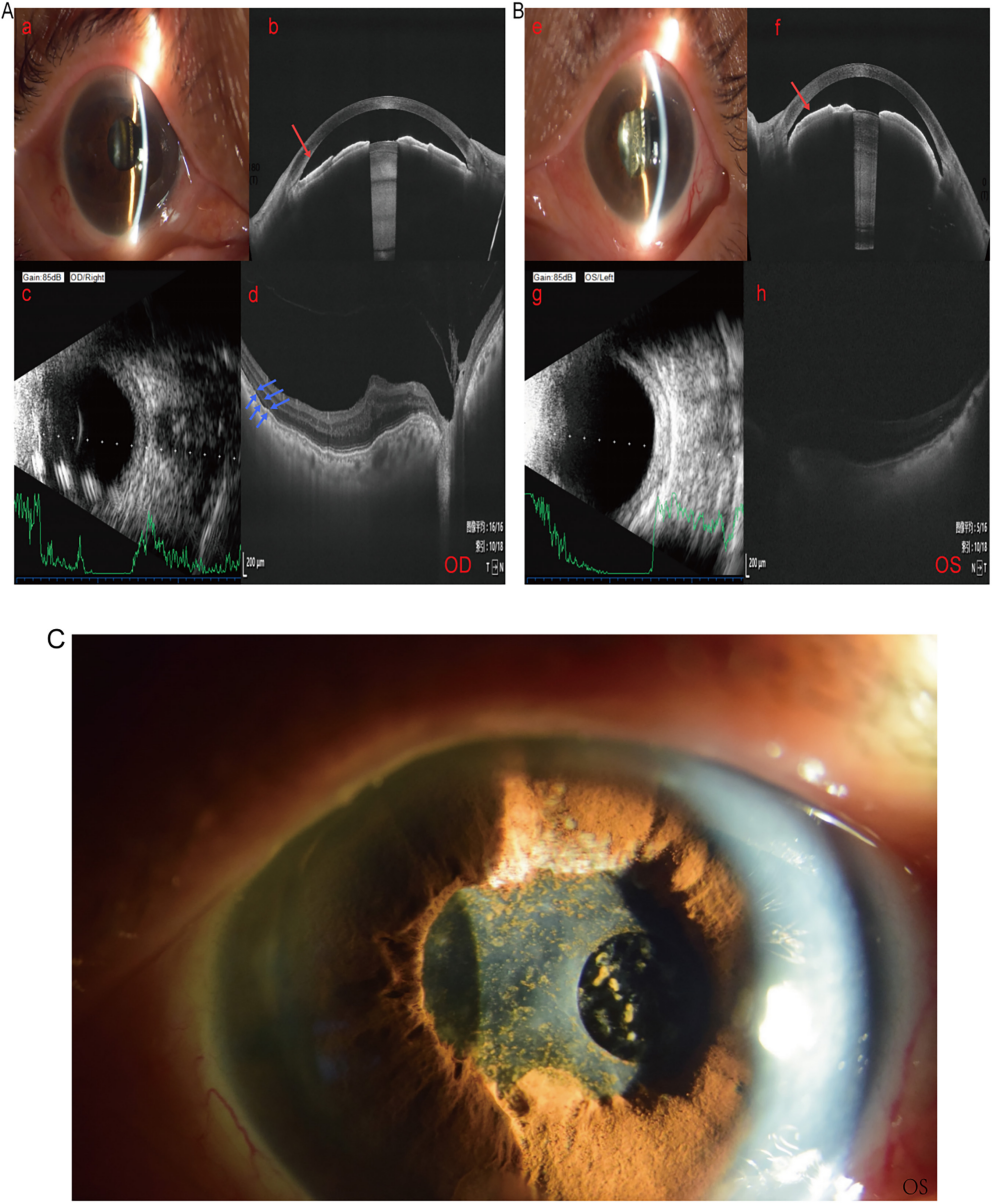
Preoperative examination and 8-month postoperative anterior segment photographs of left eye in Case 1. **(A)** Preoperative images of patient’s right eye: (a) anterior segment photograph, (b) anterior segment OCT, (c) B-scan, and (d) fundus OCT. **(B)** Preoperative left eye (e) anterior segment photography, (f) anterior segment OCT, (g) B-scan, and (h) fundus OCT, revealing bilateral lens opacity, shallow anterior chamber (red arrows), flattened fovea, and posterior vitreous detachment in the right eye with separation of the retinal pigment epithelium from the neural retina (blue arrows). **(C)** Anterior segment photograph of the patient’s left eye 8 months postoperatively, showing extensive serous exudation and an organized membrane covering the anterior surface of the intraocular lens.

The patient presented with shallow anterior chambers (AC) and lens opacities in both eyes preoperatively. OCT of right eye revealed separation between the neural retinal layer and the pigment epithelial layer in the macular region (Figure 1A). OCT of the left showed loss of foveal structure but a flat retinal surface (Figure 1B). When calculating intraocular lens (IOL) power, significant discrepancies existed between the Hoffer Q and Holladay II formulas, and no corresponding power IOLs were available domestically. Therefore, we employed the SRK/T formula and devised the surgical plan based on the maximum available IOL powers in China: for the right eye, two IOLs (Eyebright A1-UV +36.00 D and Johnson & Johnson AR40e +25.5 D) were implanted into the capsular bag; for the left eye, two IOLs (A1-UV +36.00 D and AR40e +26.50 D) were implanted into the capsular bag. It can effectively correct their high hyperopia. Both eyes underwent phacoemulsification cataract extraction with IOLs implantation under general anesthesia. Routine anti-inflammatory and anti-infective treatments were administered postoperatively. Best-Corrected visual acuity (BCVA) improved significantly on the first postoperative day: right eye 0.92 logMAR, left eye 1.30 logMAR (Table 2). The central axial depth of the anterior chamber increased compared to preoperative measurements, with IOP at 12 mmHg in both the right and left eyes (Table 3). One month postoperatively, the equivalent spherical power (SE) was −1.00 D in the right eye and +3.63 D in the left eye. At 3 months postoperatively, bilateral YAG laser treatment was performed for posterior capsular opacification (PCO). Visual acuity of two eyes remained well-maintained (Table 2). Eight months postoperatively, the patient developed blurred vision in the left eye, diagnosed as a fibrotic membrane on the anterior IOL surface (Figure 1C). Immediate left eye pupillary membrane excision and pupilloplasty were performed. Postoperative blurred vision improved, with uncorrected visual acuity (UCVA) reaching 1.30 logMAR on the first postoperative day and stabilizing at 1.22 logMAR 1 month postoperatively.

**TABLE 2:** Postoperative visual recovery following cataract surgery in the three patients.

Case	Time point	UCVA (logMAR)		BCVA (logMAR)	
		OD	OS	OD	OS
Case 1	Postop day 1	0.92	1.30	0.92	1.30
	1 week	0.92	1.22	0.82	1.22
	1 month	1.0	1.22	1.0	1.22
	3 months	1.0	1.22	1.0	1.30
Case 2	Postop day 1	1.10	–	1.10	–
	1 week	0.92	–	0.82	–
	1 month	0.92	–	0.82	–
	3 months	–	–	–	–
Case 3	Postop day 1	1.40	1.40	1.22	1.22
	1 week	1.22	1.22	1.30	1.22
	1 month	1.22	1.22	1.0	1.30
	3 months	–	–	–	–

Case 2 was blind in the left eye.

**TABLE 3:** Preoperative and postoperative intraocular pressure in cataract surgery.

IOP	Case 1		Case 2		Case 3	
Eye/time point	OD	OS	OD	OS	OD	OS
Preoperative	12	14	16	T-2	17	16
Postop day 1	12	12	15	–	17	18
1 month	16	14	14	–	17	16
3 months	16	18	–	–	–	–

IOP, intraocular pressure. Case 2 was blind in the left eye.

### 2.2 Case 2

**FIGURE 2:**
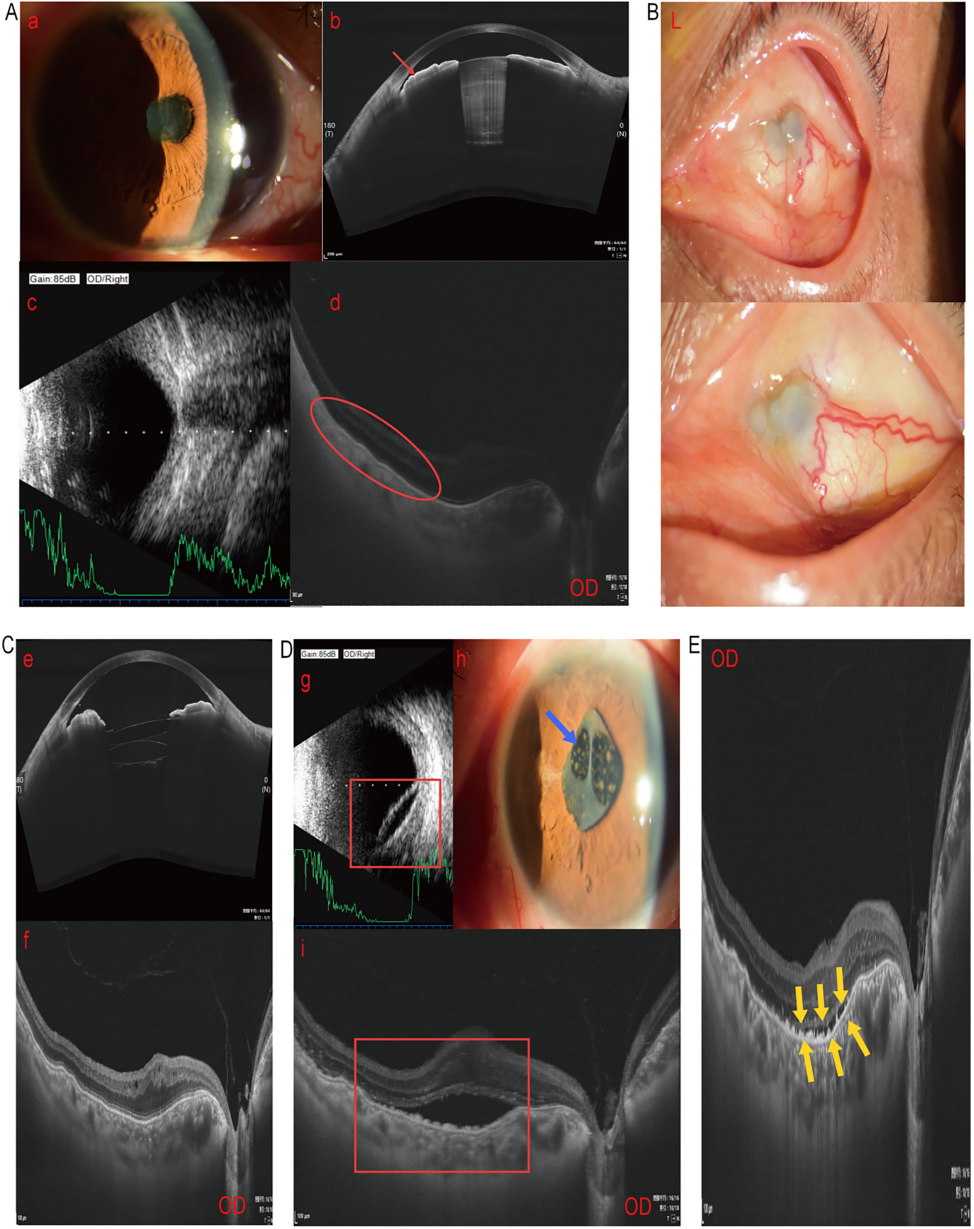
Preoperative findings and 1- and 8-month follow-up results for Case 2. **(A)** Preoperative findings (a) anterior segment photography, (b) anterior segment OCT, (c) B-scan, and (d) fundus OCT, showing right eye lens opacity and shallow anterior chamber (red arrows), with disrupted and coarse retinal ellipsoid zone reflection in the macular region of the fundus (circle). **(B)** Preoperative anterior segment photography of the left eye showing conjunctival overgrowth over the cornea with neovascular ingrowth. **(C)** One-month postoperative follow-up showing intraocular lens in position and posterior vitreous detachment. **(D)** Eight months postoperatively, (g) B-scan, (h) anterior segment photography, and (i) fundus OCT findings suggest exudative retinal detachment, subretinal fluid accumulation (rectangular frame), and a fibrotic membrane on the anterior IOL surface (blue arrow). **(E)** Fundus OCT at 1-month outpatient follow-up after anti-inflammatory treatment upon admission shows significant resolution of subretinal fluid (yellow arrows).

The patient’s general condition indicates mild anemia, urinary tract infection, and malnutrition. Ophthalmic examination revealed shallow AC with partial angle closure in the right eye; IOP 16 mmHg. Fundus OCT and B-scan showed separation of the neuroepithelial layer from the RPE layer in the macular region of the right eye, accompanied by vitreous opacities and posterior detachment (Figure 2A). The retinal nerve fiber layer thickness was 71 μm, and there was slight peripheral visual field loss. The left eye exhibited ocular atrophy with corneal conjunctivalization and neovascularization (Figure 2B). Visual evoked potentials (VEP) indicated conduction abnormalities in both eyes. We calculated IOLs power using the SRK/T formula. Under local anesthesia, we performed right eye iridotomy, phacoemulsification cataract extraction, and IOLs implantation. Intraoperatively, we implanted an A1-UV +36.00 D and an AR40e +15.00 D lens into the capsular bag. Postoperative management included routine anti-inflammatory and anti-infective therapy. On postoperative day 1, visual acuity of right improved to 1.1 logMAR (Table 2), with increased ACD and resolution of iris adhesion; IOP 15 mmHg. At the 1-month follow-up, the right eye IOL remained in place (Figure 2C), BCVA improved to 0.82 logMAR (Table 2), and the SE was +1.25 D. The patient subsequently failed to attend scheduled follow-up appointments. Eight months postoperatively, the patient was readmitted due to recurrent right eye vision decline. Examination revealed concomitant exudative retinal detachment, UES, and a fibrotic membrane on the anterior IOL surface (Figure 2D), with UCVA reduced to 1.3 logMAR. Immediate treatment included local and systemic glucocorticoid anti-inflammatory therapy supplemented with mydriasis and anti-infective measures. Following treatment, the subretinal fluid rapidly resolved. Upon discharge, UCVA had recovered to 1.0 logMAR. At the 1-month outpatient follow-up, fundus OCT demonstrated a significant reduction in the extent of the inferior retinal detachment compared to baseline (Figure 2E), with UCVA of right eye stable at 0.92 logMAR.

### 2.3 Case 3

**FIGURE 3:**
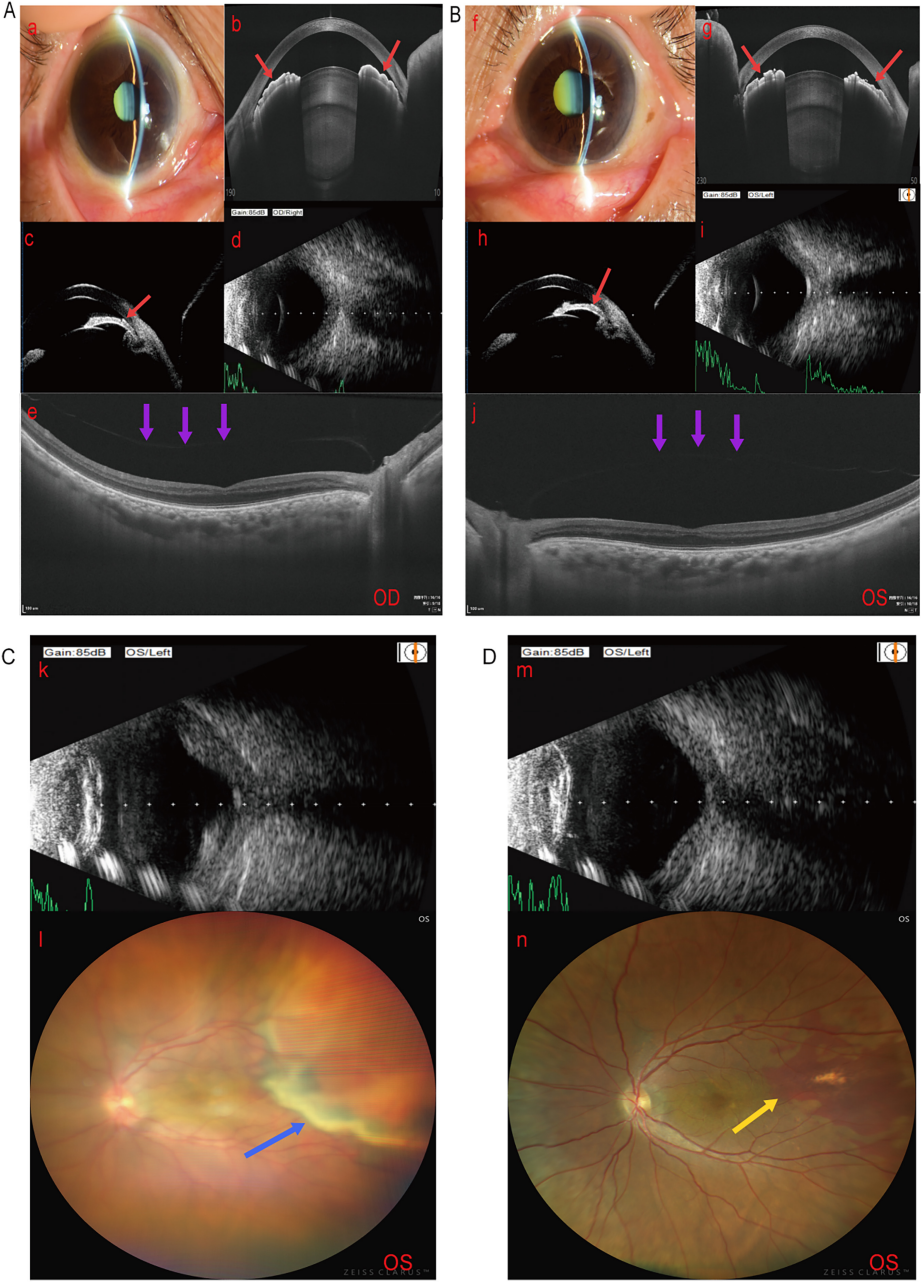
Preoperative and postoperative findings for Case 3. **(A)** Right eye (a) anterior segment photography, (b) anterior segment OCT, (c) UBM, (d) B-scan, and (e) fundus OCT. **(B)** Left eye (f) anterior segment photography, (g) anterior segment OCT, (h) UBM, (i) B-scan, and (j) fundus OCT Results indicate bilateral lens opacity, near-complete angle closure, shallow anterior chamber (red arrows), and posterior vitreous detachment (purple arrows). **(C)** Left eye on postoperative day 1 (k) B-scan and (l) fundus photography, showing localized choroidal hemorrhage and elevation (blue arrow). **(D)** One-month postoperative outpatient follow-up (m) B-scan and (n) fundus photography, demonstrating resolution of localized choroidal hemorrhage and flattened choroid (yellow arrow).

The patient presented with shallow AC bilaterally and largely closed angles. IOP was 16 mmHg in the right eye and 18 mmHg in the left eye. Fundus OCT revealed a flattened foveal center and localized hyperreflective elevation of the retinal pigment epithelium layer. The retinal neuroepithelial layer thickness was 141 micrometers in the right eye and 196 micrometers in the left eye. B-scan ultrasound indicated bilateral vitreous opacities (Figures 3A, B). Based on SRK/T formula calculations, bilateral cataract extraction with IOL implantation and iridotomy were performed under local anesthesia. Intraoperatively, an A1-UV+36.00 D IOL was implanted in the right eye, and an A1-UV+36.00 D IOL was implanted in the left eye. The right eye procedure proceeded smoothly. However, following lens extraction in the left eye, sudden intraocular pressure (IOP) elevation and shallow ACD occurred, raising suspicion of “paralytic uveitis syndrome.” Intraoperatively, viscoelastic agent was immediately injected to maintain the AC. Pericircular iridotomy was completed, followed by anterior vitrectomy with fluid aspiration and removal at 2.4–2.5 mm posterior to the corneal limbus to deepen the anterior chamber, reduce intraoperative IOP, and ensure surgical safety. Following IOL implantation, fluctuating IOP caused localized choroidal hemorrhage with retinal elevation, necessitating immediate termination of the procedure. Postoperatively, systemic corticosteroids and topical anti-inflammatory therapy were administered. On postoperative day 1, UCVA improved to 1.22 logMAR in both eyes (Table 2), IOP was 17 mmHg in the right eye and 18 mmHg in the left eye (Table 3). Ocular examination still revealed localized retinal elevation (Figure 3C). At the 1-month follow-up, the choroidal hemorrhage in the left eye had largely resolved (Figure 3D), and BCVA in both eyes stabilized at 1.0 logMAR (Table 2). The SE was +10.88 D in the right eye and +11.13 D in the left eye Subsequently; no further follow-up visits were scheduled.

## 3 Discussion

This study reports three cases of nanophthalmos combined cataract surgery in patients with high hyperopia. Case 1 presented with severe preoperative complications including ME, UES, and exudative retinal detachment. Case 2 developed UES and exudative retinal detachment in the long term due to interrupted postoperative follow-up. Case 3 experienced intraoperative ocular complications including localized choroidal hemorrhage with choroidal detachment in the left eye. Additionally, two patients who received bilateral IOLs implantation developed a fibrotic membrane on the anterior IOL surface in one eye postoperatively. Notably, one patient exhibited an extremely rare AL of only 14.54 mm among published similar cases.

Compared with normal eyes, patients with nanophthalmos are more prone to complications such as UES, ACG, and corneal endothelial cell loss following cataract surgery (19, 20). In cases of nanophthalmos with combined glaucoma, phacoemulsification combined with anterior vitrectomy and sclerectomy has demonstrated superior long-term efficacy and safety (21). Additionally, patients with nanophthalmos and ACD less than 2.5 mm experience more significant endothelial cell loss following cataract surgery, while prophylactic combined posterior scleral buckling helps reduce the risk of such complications (22). For complex nanophthalmos, combined procedures—including limited pars plana vitrectomy, anterior chamber-stabilizing phacoemulsification, IOLs implantation, and posterior capsulotomy—not only effectively control IOP, ACD, and reduce the need for antihypertensive medications, but also improve BCVA with a lower incidence of complications (23). Among the three patients reported in this article, the two with secondary glaucoma had both undergone multiple perirheidal iridectomies at other hospitals in the past; consequently, their IOP was stable within the normal range prior to surgery. Given this low-risk profile, we did not routinely perform scleral excision, which is associated with greater trauma. Preoperatively, we reviewed the patients’ existing visual fields, optic nerve fiber layer thickness, and IOP fluctuations. The results indicated that the extent of glaucomatous damage was mild in two patients. Furthermore, during postoperative follow-up, the IOP of all three patients remained stable below 20 mmHg (Table 3), and no further antihypertensive medication was required postoperatively. These observations further confirm that phacoemulsification effectively increases ACD, thereby achieving a reduction in IOP in patients with small anterior chambers complicated by ACG.

Among the three nanophthalmos patients reported in this study, two underwent cataract extraction combined with bilateral IOLs implantation, followed by intensive anti-inflammatory and anti-infective therapy postoperatively. Case 1 developed a fibrotic membrane on the anterior IOL surface in the left eye 6 months after surgery. Case 2, due to irregular postoperative follow-up, exhibited long-term complications in the right eye including a fibrotic membrane on the anterior IOL surface, UES, and exudative retinal detachment. Case 3 underwent a different surgical approach: bilateral cataract extraction with IOL implantation and iridotomy. Due to intraoperative complications, the left eye additionally underwent anterior vitrectomy. In this case, the patient received a single IOL implant and didn’t develop any of the aforementioned ocular complications postoperatively. Previous literature has reported that in cataract surgery for patients with nanophthalmos, placing two IOLs within the capsular bag may increase the risk of postoperative lens-capsular membrane formation, decreased visual acuity, and late-onset hyperopic shift (24), and YAG laser treatment is often difficult to perform and requires additional intraocular surgery (24). Therefore, when implanting two intraocular lenses, it is more advisable to use piggyback IOLs, placing one in the capsular bag and the other in the ciliary sulcus (19, 24). Similarly, studies suggest that patients with nanophthalmos, due to their limited intraocular space and size, are more suited for piggyback IOLs (19). Any residual hyperopia can be corrected through laser treatment or eyeglasses, offering potential advantages over implanting piggyback IOLs (19, 25). In our report, the two patients who received two IOLs had large lens volumes. We considered that placing one of the lenses in the ciliary sulcus could potentially lead to lens displacement, or cause the lens to rub against the iris, resulting in chronic iritis. Furthermore, friction with the iris could lead to iris pigment detachment, which could block the anterior chamber angle and exacerbate glaucoma. Therefore, during surgery, we placed both IOLs within the capsular bag to avoid these complications. However, in both of these patients, there was a fibrotic membrane on the anterior IOL surface in one eye. Some studies have reported that hydrophilic acrylic IOLs may interact adversely with glaucoma drainage valves, triggering an acute foreign body reaction that results in the formation of an inflammatory fibrous membrane on the anterior surface of IOLs (26). Other studies suggest that the fibrotic membrane on the anterior surface of the IOL after surgery is associated with the disruption of the blood-aqueous barrier and enhanced inflammatory responses, which result from a combination of surgical trauma and the patient’s own immune or vascular status (27). Additionally, multiple surgeries, incomplete membrane removal, contact between the iris and the IOL, chronic inflammation, and poor medication adherence can all lead to the development of fibrotic membrane on the anterior surface of the IOL (28). This mechanism differs from that of anterior capsular opacification (ACO). Studies indicate that ACO results from surgical trauma, which stimulates the proliferation of residual lens epithelial cells (LECs) and their migration toward the anterior capsule, where they subsequently transform into myofibroblasts, leading to white opacities at the anterior capsular opening (29, 30). In nanophthalmic eyes, the lens is typically abnormally large relative to the smaller anterior segment (high lens-to-eye volume ratio). This anatomically crowded environment may result in a higher relative density of LECs. When an anterior capsulotomy is performed during cataract surgery, the resulting surgical trauma may trigger a more intense metaplastic reaction, causing LECs to transform into myofibroblasts, which may lead to the formation of faster and thicker ACO. We hypothesize that the causes of the fibrotic membrane on the anterior surface of the IOL in the two patients reported in this article may include the following aspects: (1) The patients had small ocular volumes; even after implantation of two IOLs, anterior chamber crowding persisted, which may have compromised the stability of the blood-aqueous barrier; (2) Following disruption of the blood-aqueous barrier, inflammatory mediators and plasma proteins infiltrate the anterior chamber, potentially activating the coagulation cascade, leading to fibrin deposition and exacerbating the inflammatory response; (3) Poor medication adherence and follow-up compliance by the patients may have resulted in inadequate postoperative inflammation control. The combined effect of these factors ultimately facilitated the formation membrane associated with anterior IOL exudates.

Due to anatomical differences between nanophthalmic eyes and normal eyes, special attention is required when selecting IOLs power. Although significant progress has been made in IOLs selection for nanophthalmos, no single formula has yet been established as the optimal choice for these patients (20). Previous studies have demonstrated that the Hoffer Q and Holladay II formulas serve as useful tools for IOLs selection in patients with nanophthalmos (20). However, recent studies indicate that the Kane formula yields the most accurate results for AL ≤ 22 mm, followed by the Hill-RBF and Olsen formulas (31). For patients with shorter AL and deeper AC, the IOL power calculated using the Pearl-DGS formula provides greater accuracy (31). Another study indicates that Haigis’ formula yields more accurate results in patients with AL < 20 mm (22). However, despite the third-generation formulas (Hoffer Q, Holladay II, and SRK/T) exhibiting significant deviations from the average IOLs constant at extreme AL (32), surgeons still make patient-specific choices in clinical practice based on existing clinical evidence. Additionally, achieving emmetropia often requires IOLs with powers exceeding 34 diopters, or even 40 diopters (6). However, IOLs with powers exceeding 34 diopters are difficult to obtain in clinical practice (6). Therefore, in our report, since the new-generation formula cannot calculate IOL power for eyes with an AL < 16 mm, we comprehensively considered the patient’s ocular conditions, the error margins of various IOLs power calculation formulas, and the available IOLs power resources in China. Ultimately, we selected the SRK/T formula and obtained the highest-powered IOLs currently available domestically. Our target refractive power was emmetropia (0 D). Postoperatively, among the three cases: case 1 had an actual refractive power of −1.00 D in the right eye and +3.63 D in the left eye; case 2 had an actual refractive power of +1.25 D in the right eye; and case 3 had an actual refractive power of +10.88 D in the right eye, and the left eye measured +11.13D. All affected eyes exhibited varying degrees of hyperopic drift, with case 3 showing the most significant drift. We believe the primary reasons for these results include: (1) Limitations of the formula: The SRK/T formula lacks validated data for extremely short AL (<16.0 mm), and its predicted effective lens position (ELP) may exhibit a systematic deviation from the actual postoperative position; (2) Anatomical peculiarities of nanophthalmic eyes: An extremely shallow anterior chamber and the lens-to-axial length ratio may cause the actual IOL position to be more posterior than the predicted value, thereby resulting in hyperopic drift; (3) Upper limit on IOL power: Due to constraints on the maximum power of currently available IOLs in China, we were unable to implant IOLs with the higher powers theoretically required. Despite these refractive errors, all patients experienced a significant improvement in postoperative visual acuity, and residual refractive errors could be corrected with spectacle lenses, causing no substantial impact on patients’ daily lives.

In this study, Case 2 developed postoperative complications including ULS and exudative retinal detachment. Existing research indicates that nanophthalmos is often accompanied by extensive peripheral anterior synechiae (PAS), iris structural abnormalities, and hypoplasia of Schlemm’s canal (SC) and the trabecular meshwork (TM). These factors may collectively contribute to poor IOP control (33). Subsequently, UES develops due to scleral thickening, decreased protein permeability, and impaired venous drainage caused by collagen deposition around the choroidal veins (34). What’s more, significant fluctuations in IOP during surgery, combined with a shallow AC, may induce uveal effusion or even hemorrhage, substantially increasing surgical risks and threatening postoperative vision (23). Concurrently, the abnormally thickened sclera obstructs aqueous humor outflow through the sclera or compresses the vortex vein, leading to passive fluid accumulation in the ciliary body and choroid, which in turn triggers exudative retinal detachment (35). For this condition, conventional surgical approaches include internal jugular vein decompression, scleral incision, and partial lamellar sclerectomy, among which deep sclerectomy (with or without active drainage) has demonstrated favorable outcomes (35). Therefore, we recommend that prophylactic posterior scleral incisions be considered for high-risk patients with an AL < 16 mm and preoperative signs of choroidal thickening or foveal hypoplasia; for patients with stable intraoperative conditions and those undergoing low-energy procedures, management should be tailored to the individual.

Case 3 experienced intraoperative choroidal hemorrhage into the suprachoroidal space. Following management, no other serious complications occurred. Postoperatively, visual acuity improved in both eyes, with no additional complications noted. A study by Zheng et al. (36) indicates that patients with nanophthalmos have a significantly higher risk of choroidal hemorrhage during cataract surgery compared to conventional patients, and prolonged surgical duration further increases the risk of complications. The study emphasizes that once such complications occur, the most critical countermeasure is to immediately close the incision and terminate the surgery (36). Regarding the choice of anesthesia, Case 1 underwent general anesthesia, while Cases 2 and 3 received local anesthesia. We tend to believe that for patients with nanophthalmos who present with unique anatomical structures and higher surgical risks, general anesthesia may offer greater advantages. This approach enhances patient cooperation during surgery and facilitates continuous monitoring of vital signs, thereby reducing surgical risks to a certain extent.

Although cataract surgery for nanophthalmos increases the incidence of complications and poor visual outcomes, the widespread adoption of phacoemulsification has enhanced its safety profile (6). Multiple studies indicate that cataract surgery remains a relatively safe option for such patients (6, 21, 37). However, the success of the surgery is highly dependent on the surgeon’s extensive experience and precise calculation of the IOL power (38), as well as thorough preoperative preparation and IOL selection (39).

## 4 Conclusion

In summary, this study reports the diagnostic and therapeutic process of three patients with nanophthalmos complicated by cataracts, one of whom exhibited an AL extremely rare in adult cases. Visual prognosis following cataract surgery in patients with nanophthalmos is influenced by multiple factors. Given the high risk and severity of complications, comprehensive preoperative assessment, meticulous intraoperative technique, and standardized postoperative management are all critical. The surgeon’s experience and judgment play a decisive role in surgical safety. Developing individualized surgical and perioperative plans for these patients is essential for controlling complications and improving long-term visual outcomes.

## Data Availability

The raw data supporting the conclusions of this article will be made available by the authors, without undue reservation.
